# Near Ground Pathloss Propagation Model Using Adaptive Neuro Fuzzy Inference System for Wireless Sensor Network Communication in Forest, Jungle and Open Dirt Road Environments

**DOI:** 10.3390/s22093267

**Published:** 2022-04-24

**Authors:** Galang P. N. Hakim, Mohamed Hadi Habaebi, Siti Fauziah Toha, Mohamed Rafiqul Islam, Siti Hajar Binti Yusoff, Erry Yulian Triblas Adesta, Rabeya Anzum

**Affiliations:** 1 Department of Electrical Engineering, Faculty of Engineering, Universitas Mercu Buana, Jakarta 11650, Indonesia; galang.persada@live.iium.edu.my; 2 Department of Electrical and Computer Engineering, Kulliyyah of Engineering (KOE), International Islamic University Malaysia (IIUM), Kuala Lumpur 53100, Malaysia; habaebi@iium.edu.my (M.H.H.); rafiq@iium.edu.my (M.R.I.); sitiyusoff@iium.edu.my (S.H.B.Y.); rabeyaanzum@gmail.com (R.A.); 3 Department of Mechatronics, Kulliyyah of Engineering (KOE), International Islamic University Malaysia (IIUM), Kuala Lumpur 53100, Malaysia; 4 Department of Industrial Engineering Safety and Health, Faculty of Engineering, Universitas Indo Global Mandiri (UIGM), Palembang 30129, Indonesia; eadesta@uigm.ac.id

**Keywords:** fuzzy ANFIS, wireless sensor network, near ground, LoRa, pathloss propagation model, RSSI, jungle, forest, open dirt road

## Abstract

In Wireless Sensor Networks which are deployed in remote and isolated tropical areas; such as forest; jungle; and open dirt road environments; wireless communications usually suffer heavily because of the environmental effects on vegetation; terrain; low antenna height; and distance. Therefore; to solve this problem; the Wireless Sensor Network communication links must be designed for their best performance using the suitable electromagnetic wave behavior model in a given environment. This study introduces and analyzes the behavior of the LoRa pathloss propagation model for signals that propagate at near ground or that have low transmitter and receiver antenna heights from the ground (less than 30 cm antenna height). Using RMSE and MAE statistical analysis tools; we validate the developed model results. The developed Fuzzy ANFIS model achieves the lowest RMSE score of 0.88 at 433 MHz and the lowest MAE score of 1.61 at 433 MHz for both open dirt road environments. The Optimized FITU-R Near Ground model achieved the lowest RMSE score of 4.08 at 868 MHz for the forest environment and lowest MAE score of 14.84 at 868 MHz for the open dirt road environment. The Okumura-Hata model achieved the lowest RMSE score of 6.32 at 868 MHz and the lowest MAE score of 26.12 at 868 MHz for both forest environments. Finally; the ITU-R Maximum Attenuation Free Space model achieved the lowest RMSE score of 9.58 at 868 MHz for the forest environment and the lowest MAE score of 38.48 at 868 MHz for the jungle environment. These values indicate that the proposed Fuzzy ANFIS pathloss model has the best performance in near ground propagation for all environments compared to other benchmark models.

## 1. Introduction

In 2010, Wireless Sensor Network (WSN) technology was classified by ITU-T as a next generation network [1]. The purpose of this technological concept was to connect every sensor node using wireless technologies. This network can exchange and communicate between its nodes using wireless technologies such as WiFi, LoRa, zigbee, and many others [2]. WSN technologies have many advantages. They can monitor their environment using sensors, and their data can then be sent to the sink node and finally to the server for further processing automatically [3]. They have the ability to reduce deployment cost as they use no existing infrastructure and they incur low sensor node cost [4]. They can also extend their coverage by using ad-hoc wireless communication methods [5]. The last advantage of WSN technologies is that they can be deployed anywhere, from forests [6] to urban city environments [7]. Even though WSN technologies have many advantages, they also have disadvantages. Some of these disadvantages are small energy capacity [8], data transmission problems such as interference [9], delay [10], packet loss [11], and many others [12]. In the data transmission problem, several researchers attempted to solve it using a routing algorithm such as LEACH [13] and many others [14,15], while others tried to solve it using propagation models [16], such as the 470 MHz frequency model [17], the tunnel model [18], and many more [19,20]. 

In this study, we determine the WSN data transmission problem using an optimum propagation model that is tuned specifically for WSN. Because WSN was usually applied in isolated areas, such as in forests, jungles, and in open dirt road environments, its data transmission problem occurred mainly because of vegetation, terrain, low antenna height, and range distance. These environments usually obstruct the direct line of sight (LOS) of the electromagnetic wave energy that forms the basis of wireless data transmission between WSN nodes. The electromagnetic wave signal that was sent from the transmitter usually reached the receiver through different propagation mechanisms in a non-line of sight scenario such as diffraction, refraction, scattering, and reflection. The electromagnetic wave propagation can also be influenced by atmospheric conditions such as humidity, rain, snow, and many others [21]. This atmospheric and environmental condition can produce different electromagnetic waves from different propagation mechanisms. This phenomenon is called multipath propagation and it causes signal fading at the receiver [22]. A phenomenon such as large-scale fading happens because the magnitude of the signal strength significantly reduces as the range increases between WSN nodes. This concept is known as the pathloss propagation model between the transmitter and receiver. Moreover, in WSN, several propagation models has been developed for an accurate pathloss estimation between nodes in order to achieve power efficiency [23], quality of service of transmitted data [24], localization [25], and many other things.

Pathloss propagation models are usually divided into deterministic and semi deterministic (empirical). The deterministic model was the result of theoretical laws and principles of physics and it therefore has accurate prediction within its constraint, even though it requires detailed input that is not easy to obtain [26]. On the other hand, because the semi deterministic model combines both theory and measurement, it is less complex and easier to calculate, although the prediction accuracy may not be as high when compared to deterministic models [21]. This study aims to increase the accuracy of the semi deterministic model that can be used for WSN data transmission in the forest, jungle, and open dirt road application. We propose adding a machine learning technique in the semi deterministic models that we use. There are several machine leaning techniques that have been used to increase semi deterministic pathloss models such as RF (random forest) [27], BPNN (Back Propagation Neural Network) [28], KNN (K-Nearest Neighbour) [29], and many others [30]. In several research studies, BPNN achieves the highest accuracy with a minor difference (0.14 and 0.35) compared to SVR (Support Vector Regresion) and RF [31], while others state that ANN (Artificial Neural Network) and RF have remarkably similar performance [32]. Even though BPNN and ANN already perform well in the pathloss model, currently there are many new research efforts using the Adaptive Neuro Fuzzy Inference System (ANFIS) for pathloss modeling. Although ANFIS was another variation of ANN besides BPNN, it combines the fuzzy technique with the neural network technique [33]. This combined design approach makes ANFIS preferable compared to the single technique approach. This is motivated by its transparency in associations (reasoning to generate the membership functions and rules from a set of data training) to reach the optimal predicted pathloss value [34]. Hence, this study uses ANFIS for WSN pathloss modeling. The aim of this article is to increase the pathloss propagation model accuracy using the following planned activities below:

- Build the WSN node for the measurement experiment (LoRa radio transceiver).
- Measure the radio signal strength at jungle, forest, and open dirt road environments.
- Input those measurements into the designed ANFIS engine as the data training input.
- Build a new semi deterministic pathloss propagation model that is more accurate for jungle, forest, and open dirt road environments.
- Validate the model using RMSE and MAE against benchmark models.

## 2. Models, Materials, and Methods

In this section, we would like to explain the experimental setup that we used in this research work. The experimental setup included wireless equipment, observed environment, and measurement. After the experiment was complete, the measurement data were analyzed to provide a good pathloss propagation model in near ground wireless communication environment.

### 2.1. Related Pathloss Propagation Models

This study attempts to provide different perspectives; therefore, we will benchmark by comparing it with other popular pathloss propagation models. In this section, we consider the Okumura-Hata model that is popular for mobile communication, the FITU-R model with foliage model, and the ITU-RMA Model that has ground reflected propagation model. These three models will be compared to the Fuzzy ANFIS model to quantify the accuracy improvement achieved by the propagation model for near ground wireless communication in forest, jungle, and open dirt road environments. The benchmark models’ theoretical background is introduced below.

#### 2.1.1. Okumura-Hata Model

Okumura-Hata Model is one of the popular pathloss propagation models for mobile communication applications. This model not only considers the effects of diffraction, reflection, and scattering, but it also takes urban, suburban, and open areas into account in conjunction with carrier frequency, distance, transmitter (base station) antenna height, and receiver (mobile) antenna height. This model is based on Okumura’s measurements in Tokyo and the mathematical model by Hata [35].

(1) $$PL_{hata}\left(db\right)=69.55+\left(26.16\;\log f\right)-\left(13.82\;\log ht\right)-A\left(hr\right)+((44.9-\left(6.55*\log ht))\log d\right)$$

However, because antenna correction A(hr) was for cities, we are not using any antenna correction. In this research, we measure tropical areas near cities and open road environments. Therefore, we use Okumura-Hata for open road and suburban models:

(2) $$PL_{hata}\left(suburban\right)=69.55+\left(26.16\;\log f\right)-\left(13.82\;\log ht\right)+((44.9-\left(6.55*\log ht))\log d\right)-2{\left(\log\frac{f}{28}\right)}^{2}-5.4$$

(3) $$PL_{hata}\left(OpenRoad\right)=69.55+\left(26.16\;\log f\right)-\left(13.82\;\log ht\right)+((44.9-\left(6.55\log ht))\log d\right)-(4.78{\left(\log f\right)}^{2})+\;(18.33\;\log f)-40.94$$

where:

$PL_{hata}$ = Pathloss propagation model created by Okumura Hata.
f = Frequency carrier in MHz.
*ht* = Antenna transmitter height in meters.
  *hr* = Antenna receiver height in meters.
d = Distance between transmitter and receiver in Km.

#### 2.1.2. Optimized FITU-R Model for Near Ground Forest (Optimized FITU-R NGF) Model

Meng and colleagues proposed optimizing FITU-R (Fitted ITU-R) model in 2009 for near ground pathloss modeling in forest environment [36]. This model takes into account the plane earth model that explains direct ray in addition to ground reflected ray, which are received by the receiver, that is:

(4) $$PL_{PlaneEarth}\left(db\right)=\left(40\;\log d\right)-\left(20\;\log ht\right)-\left(20\;\log hr\right)$$

In addition, Meng also added the ITU-R foliage attenuation ($A_{\mathrm{ITU}-R\;\mathrm{foliage}}\left(db\right)$ into plane earth model, that is:

(5) $$A_{\mathrm{ITU}-R\;\mathrm{foliage}}\left(db\right)+PL_{PlaneEarth}\left(db\right)=0.2f^{0.3}d^{0.6}\left(40\;\log d\right)-\left(20\;\log ht\right)-\left(20\;\log hr\right)$$

However, using measurement data taken from oil palm tree plantation using the ITU-R Model for Near Ground Forest Model optimize, the model becomes:

(6) $$PL_{FITU-R}\left(db\right)=Af^{B}d^{C}+\left(40\;\log d\right)-\left(20\;\log ht\right)-\left(20\;\log hr\right)$$

where:

$PL_{FITU-R\;}$ = Pathloss propagation using FITU-R model.
f = Frequency carrier in GHz.
*ht* = Antenna transmitter height in meters.
*hr* = Antenna receiver height in meters.
  d = Distance between transmitter and receiver in meters.
*A*, *B*, *C* = Least squared error fit from measured data, which is 0.48, 0.43, 0.13.

#### 2.1.3. ITU-R Maximum Attenuation and Free Space Pathloss (ITU-R MA FSPL) Model

The ITU-R maximum attenuation model is recommended by the International Telecommunications Union for the frequency range 30 MHz–30 GHz. This model tells us that the ground reflected ray in this environment is negligible, since the forest ground is covered with shrubs that absorb the wave.

(7) $$PL_{MA-ITU-R}\left(db\right)=A_{M}\left(1-e^{-\frac{Rd}{A_{M}}}\right)$$

According to measurement result from Salameh, there is an indication that the direct ray is the major contributor to the received signal by the receiver which is located near the forest ground. Therefore, according to Salameh, this model is also considered as a free space model [37]. With respect to the free space model, the final Salameh model is:

(8) $$PL_{MA-ITU-R}\left(db\right)=A_{M}\left(1-e^{-\frac{Rd}{A_{M}}}\right)+32.44+\left(20\;\log d\right)+\left(20\;\log f\right)$$

where:

$A_{M}$ = Maximum excess attenuation in dB; in Salameh result was 38.
*R* = *R* is the initial slope of the attenuation curve; in Salameh result was 0.9 db/m.
d = Distance between transmitter and receiver in Km.
f = Frequency carrier in MHz.

### 2.2. Measurement Equipment

**Figure 1:**
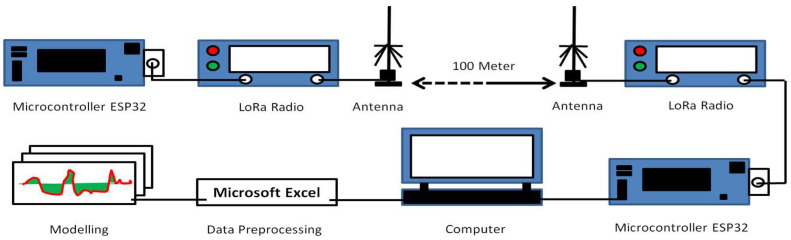
LoRa measurement equipment.

For measurement, there are several radio interfaces that are usually used for WSN communication, such as LoRa [38], Wi-Fi [39], Zigbee [25], Bluetooth [40], and many more. However, in this conducted research, we used LoRa because it can achieve long range communication [41]. In a lot of reported literature, LoRa can achieve 900 m more [42] and even more in the kilometer range [43]. For measurement, we used a LoRa transmitter and receiver pair. The transmitter was paired with microcontroller ESP32. This way, we could program the LoRa transmitter using the microcontroller to send data (packet data containing “hello” word) wirelessly to the LoRa receiver every 100 microseconds. At the receiver site, the data transmission was received by the LoRa Radio receiver. Because the data that LoRa radio received was in hexadecimal format, the microcontroller ESP32 decoded it, extracted the RSSI value, and sent those values directly to our laptop every data transmission round. Measurements were performed using walk test for every 5 m from starting point up to 100 m. The measurement data were then fed into Fuzzy ANFIS as data training, and thus provided Near Ground LoRa Electromagnetic Wave Propagation model for all observed sites. The LoRa transmitter and receiver were placed directly on top of the soil with antenna height less than 30 cm from the soil (see Figure 1). The equipment parameters’ detailed configurations are noted in Table 1.

### 2.3. Measurement Environment

**Figure 2:**
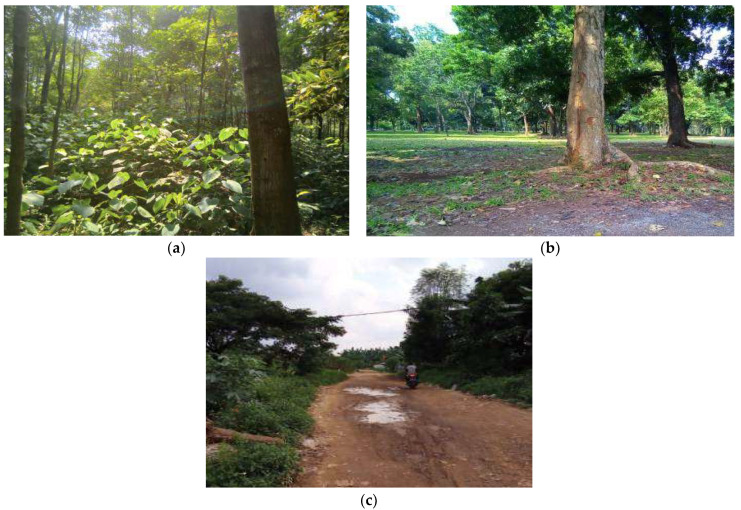
Masurement Environment, (**a**) jungle site, (**b**) forest site, (**c**) open dirt road site.

In this research’s conducted measurement, 3 different sites were chosen because of their distinct or unique properties (please see Figure 2). The first site is a jungle where overgrown mass of vegetation spreads over a large area of land. This jungle has many plants on the ground between trees and larger plants. The very dense vegetation on the ground sometimes makes it difficult or impossible for humans to go around or travel through a jungle. The second site is a forest with a dense growth of trees covering a large area of land. It has many tall trees. However, paths between trees can be traveled through by humans. Finally, the last site is an open dirt road. However, on this site, the vegetation grows on the left side and on the right side of the road. These 3 measurement sites were chosen to study the effect of their distinct or unique properties and their effect on electromagnetic wave propagation. It will provide us with more information about electromagnetic wave behavior that propagates near the ground. The measurements were carried out in 3 different locations with 3 distinct characteristics. In all environments, the measurements were carried out with 54 different LoRa radio configurations. The forest environment, located in the eastern suburbs of Jakarta, has a GPS coordinate −6.366020, 106.901294. The jungle environment is also located in the eastern suburbs of Jakarta, and it has GPS coordinates −6.358641, 106.903730. The open dirt road environment is located in the western suburbs of Jakarta at GPS coordinates −6.214478, 106.747576.

### 2.4. Adaptive Neuro Fuzzy Inference System Method

**Figure 3:**
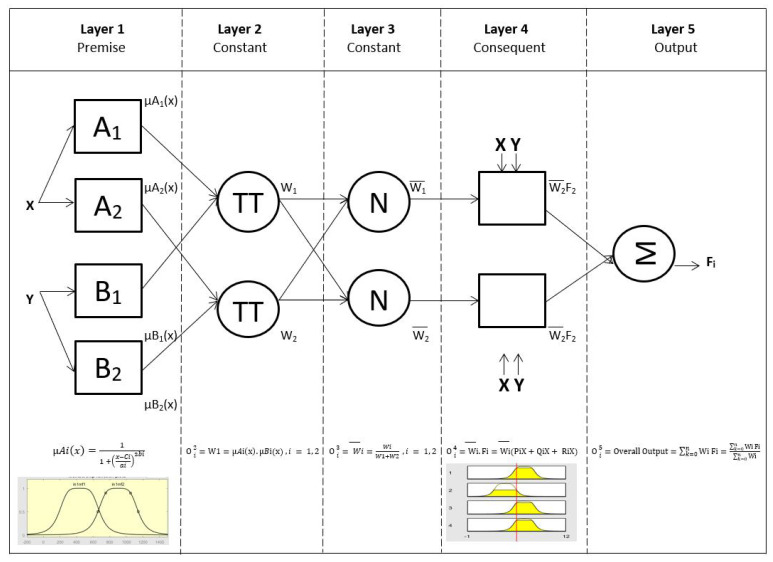
ANFIS architecture.

Fuzzy system is mainly used for control, such as controlling robot movement [44,45], speed [46], and other factors [47,48,49]. However, fuzzy system can basically be used for anything. Adaptive Neuro Fuzzy Inference System (ANFIS) architecture based on Jang’s research consists of five stages [41,42], as shows in Figure 3. The square-shaped nodes show adaptive nodes, while the circular shapes are the fixed nodes.

The Fuzzy ANFIS architecture based on Jang’s research can be seen in this equation: 

For stage one, each output is symbolized by $O\begin{array}{c} 1\\ i \end{array}$, which serves to raise the degree of membership.

(9) $$O\begin{array}{c} 1\\ i \end{array}=µA_{i}\left(x\right)\;\mathrm{and}\;O\begin{array}{c} 1\\ i \end{array}=µB_{i}\left(x\right),\;i=1,\;2$$

where:

*i* = every node in Fuzzy ANFIS architecture.
*x =* is the input to node *i.*
*A*, *B =* is the linguistic label (such as small, large, etc.).

In this stage, every membership function type can be used, but in Jang’s method [50], generalized bell membership functions were used to provide two outputs: maximum equal to 1 and minimum equal to 0. Therefore, we obtain:

(10) $$µAi\left(x\right)=\frac{1}{1+{(\frac{x-c_{i}}{a_{i}})}^{2*b_{i}}}$$

where:

a, b, c = is the parameter set.

The second stage is constructed by multiplying the two input signals. Every node represents the firing strength of fuzzy inference.

(11) $$O\begin{array}{c} 2\\ i \end{array}=µA_{i}\left(x\right).\;µB_{i}\left(x\right),\;i=1,\;2$$

For the next stage, normalization was applied for each firing of fuzzy inference.

(12) $$O\begin{array}{c} 3\\ i \end{array}=W_{i}=\frac{W_{i}}{W1+W2}\;,\;i\bar{=}1,\;2$$

where:

W = is the firing strength of node.
$\bar{W}$ = is the normalized firing strength of node.

The next stage contains the calculation of the output based on the parameters of the rule consequent.

(13) $$O\begin{array}{c} 4\\ i \end{array}=W_{i}\;.F_{i}=W_{i}.\left(\bar{P_{i}x}+Q_{i}x\;+R_{i}x\right),\;i\;=1,\;2$$

where:

P,Q,R = is the parameter set.

Finally, the last stage computes the overall output as the summation of all input signals.

(14) $$O\begin{array}{c} 5\\ i \end{array}=\mathrm{Overall}\;\mathrm{Output}=\sum_{k=0}^{n}W_{i}\;.F_{i}=\frac{\sum_{k=0}^{n}W_{i}\;.F_{i}}{\sum_{k=0}^{n}W_{i}}$$

Because ANFIS learns from gradient descent and chain rule, error rate needs to be known for data training for each node output. Assuming i-th position node outputs as $O_{i}$, the training data set has P number of entries and the error function can be measured as:

(15) $$E_{p}=\overset{\#L}{\underset{m=1}{\sum }}{\left(T_{\mathrm{mp}}-\;O\begin{array}{c} L\\ mp \end{array}\right)}^{2}$$

where:

$E_{p}$ = is error measure which is the sum of squared errors.
$T_{mp}$ = is *m* component from *P* output target vector.
$O\begin{array}{c} L\\ mp \end{array}$ = is *m* component from actual output vector that has been produced by *P* input vector.

Hence, the error rate can be calculated as:

(16) $$\frac{\partial E_{p}}{\partial O\begin{array}{c} k\\ i\;p \end{array}}=\overset{\#k+1}{\underset{m=1}{\sum }}\frac{\partial E_{p}}{\partial O\begin{array}{c} k+1\\ m\;p \end{array}}\;\frac{\partial O\begin{array}{c} k+1\\ m\;p \end{array}}{\partial O\begin{array}{c} k\\ i\;p \end{array}}$$

where 1 ≤ *k* ≤ L−1 is error rate of an internal node; it is expressed as linear combination error rate of nodes in the next stages. Therefore, for all 1 ≤ *k* ≤ L and 1 ≤ *i* ≤ #(*k*), we can find $\frac{\partial Ep}{\partial O\begin{array}{c} k\\ i\;p \end{array}}\;$, using Equations (15) and (16). Now, we have α as a parameter of the adaptive network.

(17) $$\frac{\partial E}{\partial \alpha }=\underset{O*\epsilon S}{\sum }\frac{\partial Ep\;*}{\partial O\;*}\frac{\partial O}{\partial \alpha }$$

where:

*S* = shows the set of nodes whose output depends on α.

Derivative for overall error measurement E with respect to α is:

(18) $$\frac{\partial E}{\partial \alpha }=\sum_{p=1}^{p}\frac{\partial Ep}{\partial \alpha }$$

Therefore, we can write the updated formula for generic parameter α as follows:

(19) $$\Delta \alpha \;=-\eta \frac{\partial E}{\partial \alpha }$$

where:

*η* = is a learning rate.

The learning rate can be written as

(20) $$\eta =\frac{k}{\sqrt{\sum_{\alpha }\;{\left(\frac{\partial E}{\partial \alpha }\right)}^{2}}}$$

where:

*k* = is the step size of length of each gradient transition in the parametric space.

According to Zadeh, in 1975, fuzzy foundation was developed from Linguistic Variable and its Application to Approximate Reasoning [51]. Using those foundations, we can say that fuzzy rule was developed in order to model the qualitative aspects of human expertise (reasoning based on experience) [52] and solve or adapt the problem [53]. Therefore, using Equations (10) and (14), we can write the propagation model for near ground wireless communication in forest, jungle, and open dirt road environment as: $\frac{\sum_{k=0}^{n}\frac{1}{1+\left[{(\frac{x-c_{i}}{a_{i}})}^{2*b_{i}}\right]}\frac{1}{1+\left[{(\frac{x-c_{i}}{a_{i}})}^{2*b_{i}}\right]}Fi\;}{\sum_{k=0}^{n}\frac{1}{1+\left[{(\frac{x-c_{i}}{a_{i}})}^{2*b_{i}}\right]}\frac{1}{1+\left[{(\frac{x-c_{i}}{a_{i}})}^{2*b_{i}}\right]}}$

where:

x = Input variable such as frequency, bandwidth, spreading factor, range, and others.
*a* = Defines the width of the membership function input.
b = Defines the shape of the curve on either side of the midland.
c = Defines the center point of the membership function.
Fi = Constant Output Level generated automatically by ANFIS.

## 3. Results and Discussion

Our initial measurement result shows varying signal strength for each measurement point and each radio configuration. This problem we encountered was caused by different propagation phenomena such as diffraction, refraction, and reflection of the transmitted signal because of the surrounding vegetation environment [54]. As stated in Section 1, in the jungle, forest, or open road environment, vegetation and other things can induce small-scale multipath fading for the electromagnetic wave. However, as we observe the multipath fading in the dense jungle, the jungle is not as static as it may seem. The spatial distribution component of the multipath loss was clearly evident in the measured values collected at different points in the jungle. The temporal distribution component of the multipath loss in the jungle was also present in the fluctuation of the received signal strength at any point. This fluctuation is due to the dynamic movement of hidden obstacles on the jungle floor, tree trunks, canopies, and treetops. The jungle is rich with livestock of birds, squirrels, monkeys, rats, and many other living animals, in addition to the humans conducting the experiments. If we also add this factor, the effect of occasional wind can also result in detectable fluctuation in the RSSI. As expected, the multipath temporal distribution component is not as severe as it is in a typical urban environment. Evidence of this environmental dynamicity can be captured using a highly sensitive microphone or high frame rate camera. Therefore, to make the measurements reliable, we transmit 10 packet data and perform averaging for its signal strength. Even though we performed averaging for each measurement point, we still found that every measurement point also had a varying measurement result from other nearby measurement points. This was reflected in the zig-zag measurement chart for every environment.

**Figure 4:**
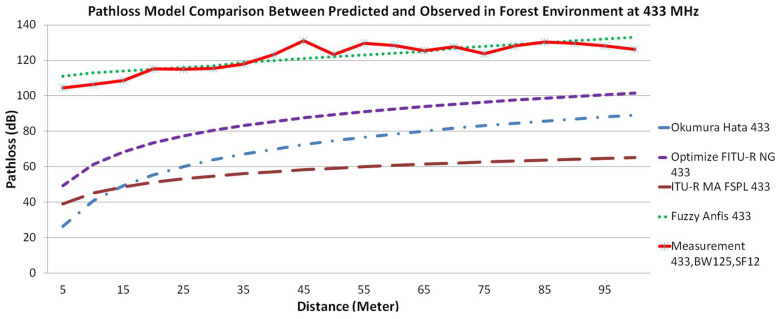

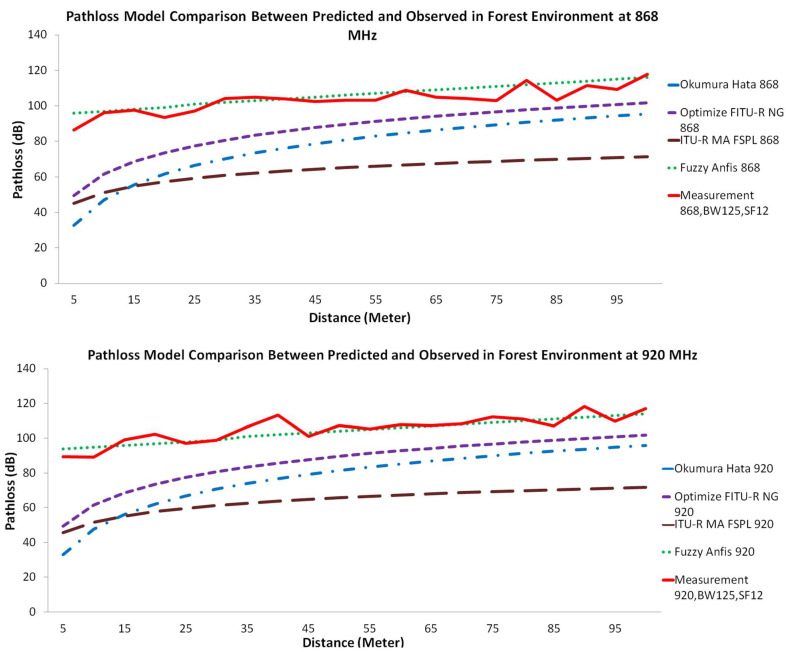
Pathloss model comparison between predicted and observed value in forest environment for frequency carrier (**top**) 433 MHz, (**middle**) 868 MHz, and (**bottom**) 920 MHz.

**Figure 5:**
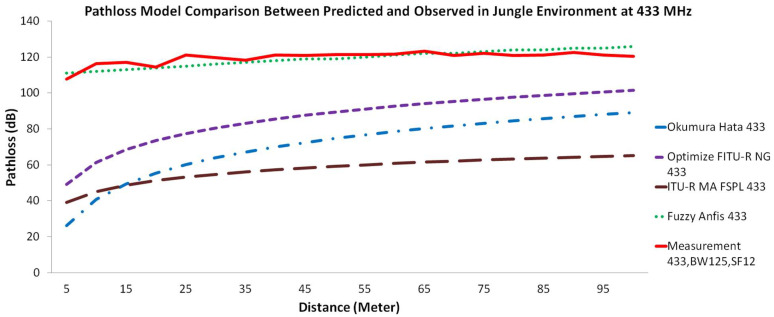

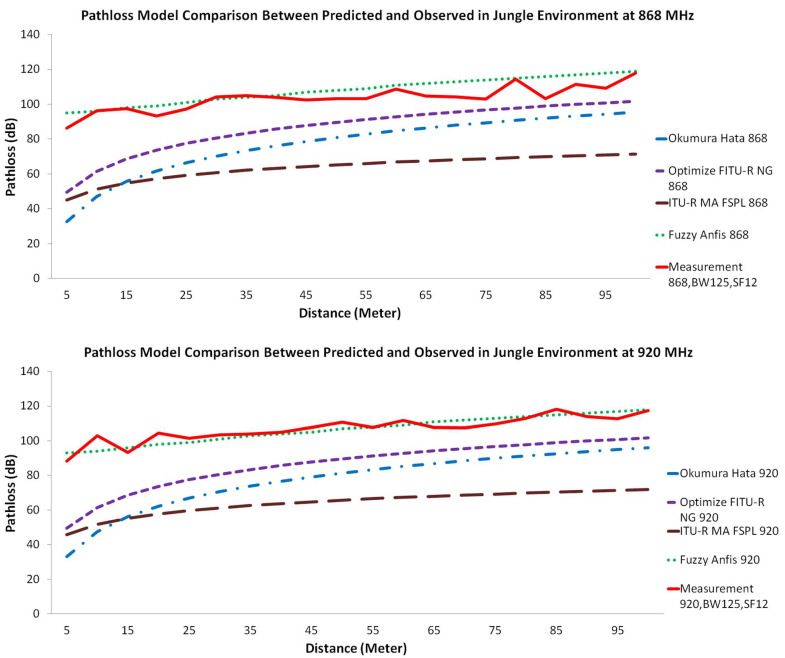
Pathloss model comparison between predicted and observed value in jungle environment for frequency carrier (**top**) 433 MHz, (**middle**) 868 MHz, and (**bottom**) 920 MHz.

**Figure 6:**
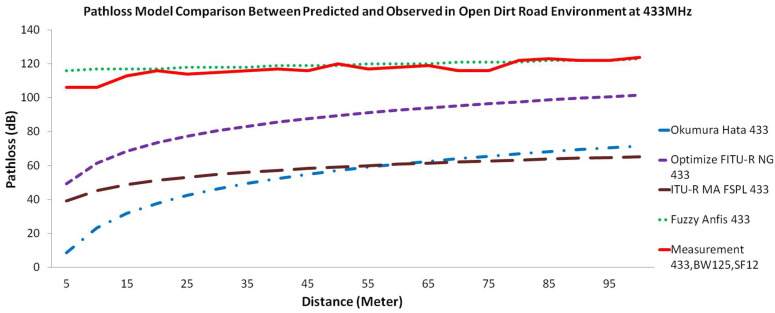

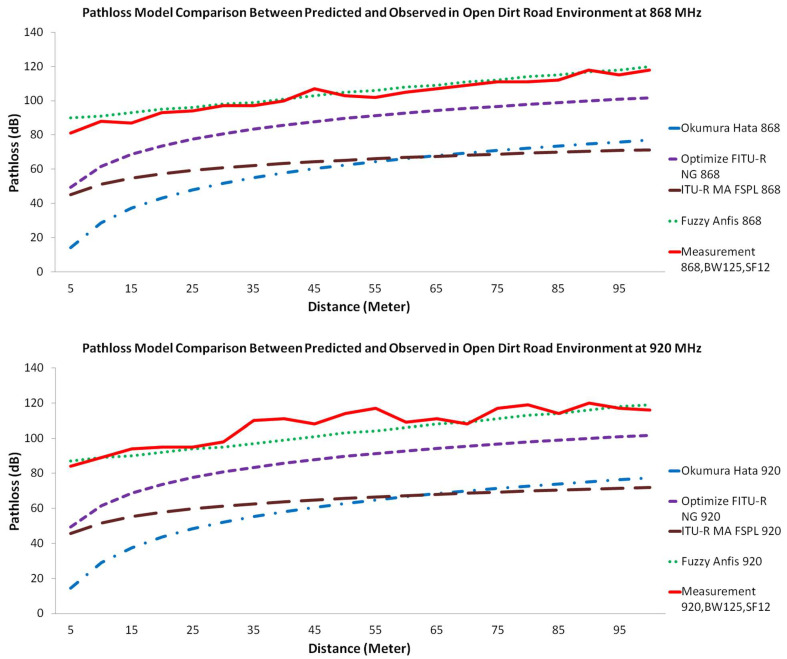
Pathloss model comparison between predicted and observed value in open dirt road environment for frequency carrier (**top**) 433 MHz, (**middle**) 868 MHz, and (**bottom**) 920 MHz.

In this study, we present a comparison for every respected near ground pathloss propagation model using three different frequency bands. Figure 4 illustrates the comparison of the different pathloss models with measurements in the forest environment. Figure 5 illustrates the comparison of the pathloss models with measurements in the jungle environment, while Figure 6 compares the models with measurements in the open dirt road environment. In all figures, measurement results are plotted with a red solid line, the Okumura-Hata model is presented with a blue dashed-dotted line, the Optimized FITU-R Near Ground model is presented with a violet dashed line, ITU-R Maximum Attenuation Free Space model is presented with a brown dashed line, and the Fuzzy ANFIS model is presented with a green dotted line.

If we carefully observe Figure 4, Figure 5 and Figure 6, there are a few interesting facts. At first, even though the jungle has a dense vegetation environment in comparison to the open dirt road, its pathloss measurement shows better results. Consider the rule of thumb that decreasing the transmit frequency leads to a reduced pathloss; this is evident in the open dirt road and forest environments. As shown in Figure 6, there is less reflection and scattering, due to foliage taking place. However, as the environment changes to severe scattering and multiple reflection conditions in the jungle, this rule of thumb is somewhat violated, especially for the 433 MHz frequency band. Note that the shrub height from the jungle floor is approximately 1 m, as shown in Figure 2a. Since the transmitter and receiver antenna heights are only 30 cm from the jungle floor, they suffer from what is known as the Fresnel zone non clearance effect [55]. As the antenna is trapped underneath and in between the taller shrubs, this effect results in a further pathloss component caused probably by diffraction at both transmitter and receiver ends and severe scattering from the taller shrubs (resembling diffraction from a roof-top or lamb-post antenna in a city environment). Due to the larger wavelength size, and hence larger Fresnel zone diameter, this diffraction pathloss component is magnified at the lower frequency of 433 MHz, as confirmed by [55] and compared to 868 and 920 MHz bands. Furthermore, due to this dense vegetation on the jungle floor, the signal travels several shrub-edge diffraction points to reach the receiver antenna trapped under the shrubs on the other side of the jungle floor. These multiple diffraction components have weakened the signal strength and added to the overall link pathloss. Because both the transmitter and receiver were placed above the ground with only 30 cm height, we assume that in the jungle environment, most of the LoRa signal ground reflections were absorbed by wet grass on the floor, resulting in small ground reflection. On the contrary, there are stronger reflections for LoRa signals in the open dirt road environment because the road was not covered by wet grass.

Another interesting fact is the large difference between the measurement and the predicted empirical pathloss model. The example of this was the measurement of 5 m using the 868 MHz band. Based on the empirical model ITU-R MA FSPL at 5 m, the RSSI value should be at −29 dBm while in the real measurement, it is −66 dBm (a −37 dB difference). This is because the ITU-R MA FSPL empirical model was derived from free space pathloss. Thus, we can explain this phenomenon using Chyriskos formulation which states that “signal loss is the sum of two independent attenuation processes: free space loss and losses due to obstacles” [56]. The RSSI value for free space pathloss using the 868 MHz frequency band at 5 m was −25 dBm, thus −41 dB was contributed by heavy obstacle loss. There are two main components for this obstacle loss, as follows:

- Obstacle loss due to the vegetation environment that obstructed the signal. As stated by Salameh, “direct ray is the major contributor to the received signal by the receiver which is located near the ground of the forest. The implication here is that the ground reflected ray in this environment is negligible, since the forest ground is covered with shrubs that can absorb the wave” [37].
- Obstacle loss due to the Fresnel zone that was caused by low antenna height. Because our measurement was placed with an antenna height of less than 30 cm, this Fresnel zone acted as an obstacle according to Adi and Kitagawa. They stated that the Fresnel zone area is influenced by antenna height: the higher the value of the antenna height, the greater the percentage of the Fresnel zone clearance [55]. They further state that the lower frequency causes a bigger Fresnel zone; thus, it is no wonder that the measurement on 433 MHz generated a lower RSSI signal compared to 868 MHz and 920 MHz.

If we carefully observe the Okumura-Hata pathloss propagation model, we find that its behavior does not match the measurement results. The presented results in Figure 4 through Figure 6 show that the Okumura-Hata model has a curved line with between 5 and 50 m between the transmitter and receiver. Although the measurement results’ trendline is a zig-zag line, overall measurement behavior does not show any curved shape anywhere. Moreover, there is a gap in the predicted values between Figure 4 and Figure 5, compared to Figure 6. This difference is assumed to occur because of the nature of the model. To predict the loss value in the forest and jungle, we used the Okumura-Hata suburban model, while in the open dirt road environment we used the Okumura-Hata open area model. Using the Okumura-Hata suburban model results in a high difference of under-estimation between the observed values and the predicted values. The differences for the lowest value come from 868 MHz with 11 dB delta at 85 m for the forest and jungle. The difference for the highest value comes from 433 MHz and is 78 dB delta and 81 dB delta both at 5 m distance for the forest and jungle. However, if we use the Okumura-Hata open area model, the under-estimation difference would be even higher. The difference for the lowest value coming from 868 MHz is 38 dB delta at 55 m while for the highest value coming from 433 MHz is 97 dB delta at 5 m. Coming to the second model, which is the Optimized FITU-R Near Ground model, this model also presents a curved line between 5 and 70 m from the transmitter and receiver. This model behavior indicates that the model does not match with the measurement results. The Optimized FITU-R Near Ground model under-estimates the pathloss also by a high difference from the observed values. The difference for the lowest value comes from 868 MHz with 4 dB delta at 85 m for the forest and jungle and 11 dB delta for the open dirt road at 55 m. The difference for the highest value coming from 433 MHz is 55 dB delta, 59 dB delta, and 57 dB delta at 5 m for all environments. For the third model, the ITU-R maximum attenuation free space model, it shows a curved line between 5 m and 85 m from the transmitter and receiver. This model behavior indicates that the model does not match the behavior of the measurement results. However, compared to both the Optimized FITU-R Near Ground and Okumura-Hata models, this model closely mimics the measurement trendline behavior but it still under-estimates with a large difference from the observed values. The difference for the lowest value coming from 868 MHz is with 33 dB delta at 85 m for the forest and jungle and 32 dB delta for the open dirt road at 15 m. The difference for the highest value coming from 433 MHz is 73 dB delta at 45 m for the forest, 71 dB delta at 10 m for the jungle, and 67 dB delta at 5 m for the open dirt road. For the proposed Fuzzy ANFIS, although it is not identical, this model is the most accurate model that mimics the measurement trendline and matches the observed values’ behavior. This model has a low difference between observed values with predicted values. The difference for the lowest value comes from 433 MHz with 0 dB delta at 20 m for the forest and jungle and 433 MHz 0 dB delta at 90 m for the open dirt road. The difference for the highest value coming from 920 MHz with 11 dB delta at 40 m for the forest, 868 MHz with 11 dB delta at 75 m for the jungle, and 920 MHz 13 dB delta at 35 m for the open dirt road. To validate the results, we need to validate the analysis using statistical evaluation tools. Bakinde et al propose RMSE and MAE for the error statistical evaluation to be used in pathloss prediction analysis [57]. Table 2 shows the statistical evaluation for each pathloss propagation model using RMSE while Table 3 shows us statistical evaluation for each pathloss propagation model using MAE.

The statistical error analysis concept tells us that the smallest value indicates that the model has the best matching performance between the predicted values and observed values. We can see that the best model based on statistical evaluation is fuzzy ANFIS with the lowest RMSE score of 0.88 at 433 MHz for the open dirt road environment and the lowest MAE score of 1.61 at 433 MHz also for the open dirt road environment. The second-best model would be Optimized FITU-R Near Ground with the lowest RMSE score of 4.08 at 868 MHz for the forest environment and the lowest MAE score of 14.84 at 868 MHz for the open dirt road environment. The third best model would be Okumura-Hata with the lowest RMSE score of 6.32 at 868 MHz for the forest environment and the lowest MAE score of 26.12 at 868 MHz also for the forest environment. The worst model would be the ITU-R Maximum Attenuation free space with the lowest RMSE score of 9.58 at 868 MHz for the forest environment and the lowest MAE score of 38.48 at 868 MHz for the jungle environment. 

## 4. Conclusions

In this study, we investigated and analyzed the behavior of LoRa pathloss propagation models in the near ground with low transmitter and receiver antenna heights from the ground. Furthermore, we introduced a fuzzy ANFIS model to predict the near ground pathloss in different tropical environments. We can see from the presented results that the most accurate prediction model, that agrees with the measurement results, in forest, jungle, and open dirt road environments, is the proposed fuzzy ANFIS model. We validated the performance using RMSE and MAE statistical analysis tools. The fuzzy ANFIS model achieves the lowest RMSE score of 0.88 at 433 MHz for the open dirt road environment and the lowest MAE score of 1.61 at 433 MHz also for the open dirt road environment. The Optimized FITU-R Near Ground model achieves the lowest RMSE score of 4.08 at 868 MHz for the forest environment and the lowest MAE score of 14.84 at 868 MHz for the open dirt road environment. The Okumura-Hata model achieves the lowest RMSE score of 6.32 at 868 MHz for the forest environment and the lowest MAE score of 26.12 at 868 MHz also for the forest environment. The ITU-R Maximum Attenuation Free Space model achieves the lowest RMSE score of 9.58 at 868 MHz for the forest environment and the lowest MAE score of 38.48 at 868 MHz for the jungle environment. This RMSE and MAE values indicate that the ITU-R Maximum Attenuation Free Space model is not suitable in predicting RSSI value for near ground propagation for all environments, while fuzzy ANFIS has the most accurate prediction of RSSI values and pathloss for near ground propagation for all environments.

## Figures and Tables

**Table 1:** LoRa setting.

Parameter	Value
Frequency	433, 868, 920 MHz
Bandwidth	125, 250, 500 KHz
Spreading Factor	7–12
Antenna Gain	0 dBi
Tx-power	20 dbm
Measurement Parameter	RSSI

**Table 2:** Statistical evaluation for each pathloss propagation model using RMSE.

Measurement		Propagation Model			
Environment	Frequency	Okumura-Hata	Optimized FITU-R NG	ITU-R MA FSPL	Fuzzy ANFIS
Forest	433 MHz	12.28	8.57	15.49	1.30
	868 MHz	6.32	4.08	9.58	1.46
	920 MHz	6.67	4.55	15.79	1.23
Jungle	433 MHz	11.97	8.18	15.28	1.11
	868 MHz	6.45	4.16	9.82	1.91
	920 MHz	7.20	5.03	16.35	1.06
Open Dirt Road	433 MHz	22.77	10.56	22.29	0.88
	868 MHz	16.22	5.99	15.49	0.98
	920 MHz	17.48	7.40	16.72	1.66

**Table 3:** Statistical evaluation for each pathloss propagation model using MAE.

Measurement		Propagation Model			
Environment	Frequency	Okumura Hata	Optimized FITU-R NG	ITU-R MA FSPL	Fuzzy ANFIS
Forest	433 MHz	51.44	36.11	65.02	3.05
	868 MHz	26.12	16.89	39.94	3.80
	920 MHz	28.20	19.45	42.02	3.30
Jungle	433 MHz	50.98	30.98	60.31	3.35
	868 MHz	29.16	15.00	38.48	5.13
	920 MHz	32.59	18.90	41.89	3.10
Open Dirt Road	433 MHz	46.48	24.54	57.86	1.61
	868 MHz	40.80	14.84	42.33	2.31
	920 MHz	43.63	18.07	45.07	4.05

## Data Availability

Not applicable.
